# Opium poisoning following self‐medication of radiation‐induced dermatitis with topical use of opium latex traditional extract; a teaching case

**DOI:** 10.1002/ccr3.4661

**Published:** 2021-08-16

**Authors:** Mansoureh Dehghani, Seyyed Morteza Hosseini, Sara Molkara, Danial Fazilat‐Panah, Omid Mehrpour, Davood Soroosh, Elham Zarei, James S. Welsh, Mohammad Nematshahi, Seyed Alireza Javadinia

**Affiliations:** ^1^ Cancer Research Centre Neyshabur University of Medical Sciences Neyshabur Iran; ^2^ Department of Anesthesiology and Critical Care Neyshabur University of Medical Sciences Neyshabur Iran; ^3^ Department of Dermatology Neyshabur University of Medical Sciences Neyshabur Iran; ^4^ Cancer Research Center Babol University of Medical Sciences Babol Iran; ^5^ Medical Toxicology and Drug Abuse Research Center (MTDRC) Birjand University of Medical Sciences (BUMS) Birjand Iran; ^6^ College of Public Health Mel and Enid Zuckerman University of Arizona Tucson AZ USA; ^7^ Department of Internal Medicine Sabzevar University of Medical Sciences Sabzevar Iran; ^8^ Student Research Committee Mashhad University of Medical Sciences Mashhad Iran; ^9^ Edward Hines Jr VA Hospital and Loyola University Chicago Stritch School of Medicine Chicago IL USA; ^10^ Department of Anesthesiology and Critical Care Sabzevar University of Medical Sciences Sabzevar Iran; ^11^ Clinical Research Development Unit Hospital Research Development Committee Sabzevar University of Medical Sciences Sabzevar Iran

**Keywords:** opium latex traditional extract, opium poisoning, radiation‐induced dermatitis, self‐medication

## Abstract

Despite Radiation‐induced dermatitis is a self‐limiting complication, it can be complicated if inappropriate self‐medications have been used such as opium latex traditional extract.

## Graphical abstract

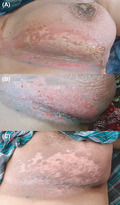

## 1 INTRODUCTION

Radiation‐induced dermatitis is the most prevalent side effect of radiotherapy regardless of the region of treatment. We present the case of a breast cancer patient who self‐treated her radiation‐induced dermatitis by opium latex traditional extract and ended up hospitalized for opium overdose and dermatological bacterial infection.

The most common acute side effect of external beam radiotherapy is “radiation dermatitis”.^2^ Nearly all women who receive external beam radiotherapy for breast cancer, experience some degree of dermatitis. The acute phase is predominantly defined as occurring within 30–90 days of radiation exposure.^4^ Despite substantial developments in radiotherapy techniques, effective preventive care is still lacking, and current evidence does not provide adequate guidelines for managing acute skin reactions.^7^ Different institutions and experts prescribe various topical products. This report presents a case of a breast cancer patient who self‐treated her radiation dermatitis and ended up hospitalized for opium overdose and her breast skin became infected.

## 2 CASE

**FIGURE 1:**
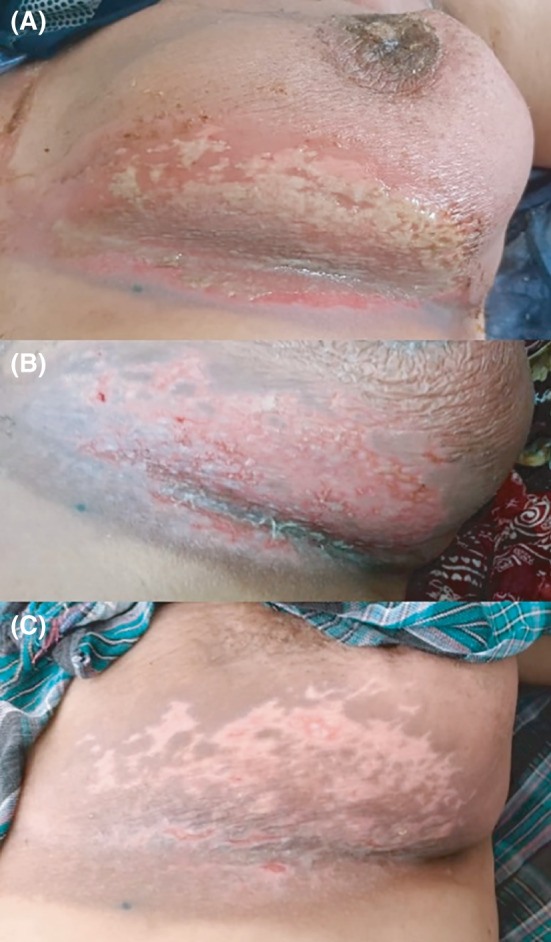
Radiation‐induced dermatitis complicated by a bacterial infection (A), recovery from infection one week after empirical antibiotic treatment (B), dermatitis healing three weeks after treatment rest

This report presents a case of a 43‐year‐old female patient (height: 155 cm, weight: 72 kg) with known T2N1, hormone receptor‐positive, Her2 negative breast cancer (ki67: 10%). The patient underwent breast‐conserving surgery and axillary lymph node dissection. She was then referred to the oncology clinic for adjuvant therapy, where she received AC‐T (dose‐dense doxorubicin plus cyclophosphamide followed by weekly paclitaxel) chemotherapy regimen. Whole‐breast and axillary radiation treatment was initiated afterward, aiming to treat the target volume to a total dose of 50 Gy along with a 10 Gy tumor bed boost. At the cumulative dose of 40 Gy in 20 fractions, erythema (grade one radiation dermatitis) gradually appeared on skin in the axillary region and inframammary fold. The dermatitis progressed to grade three at 50 Gy (before the boost to the surgical lumpectomy bed). During this period, conservative treatment and Alpha ointment (Lawsonia inermis +Curcuma longa +Pastacia terebinth, Exir Danesh Asia Pharmaceutical Co, Iran) were administered but the response was suboptimal. Therefore, after completing the whole‐breast irradiation portion (50 Gy in 25 fractions) but before the boost phase, a 3‐week treatment rest was planned to allow the dermatitis to heal. One week later, a consultation was requested from the hospital emergency department regarding our patient. Apparently, she had applied opium extract the night before as a self‐treatment/home remedy and was admitted to the hospital due to what appeared to be opium poisoning. This diagnosis was based on her clinical presentation, which included lethargy, rigidity, bradycardia, oxygen saturation decline, and bilateral miosis. The patient had applied the extract on all breast and axillary regions with dermatitis. She was treated accordingly with antidote administration and supportive care and was discharged the next day. Opium‐induced toxicity can be diagnosed clinically based on the patient history, physical examination and the response to naloxone; all of these were taken into consideration and were conclusive in the presented case. The next day in the clinic, follow‐up examination detected noticeable bacterial infection on the skin area that was treated by topical opiate ointment (Figure 1A). After consultation with the infectious disease specialist, the patient was treated with glindamycin (900 mg intravenously and 300 mg oral, every 3 hours) and gemifloxacin (320 mg daily oral) for one week. Although the infection ameliorated (Figure 1B), unhealed dermatitis still persisted, necessitating one more week of rest. In the end, the patient recovered from dermatitis with post‐inflammatory hypopigmentation (Figure 1C). The boost phase of radiotherapy was then continued without any further complication.

## 3 DISCUSSION

Radiotherapy has been extensively used in breast cancer treatment to reduce the risk of loco‐regional recurrence in an adjuvant setting after lumpectomy or after mastectomy.^1^ The most common transient, acute side effect of radiotherapy is "radiation dermatitis" or, radiation‐induced injury of the skin. This reaction occurs in approximately 95 percent of patients receiving radiotherapy,^2^ particularly skin cancer, breast cancer, and head and neck cancer,^3^ in which more radiation dose is deposited at the patient skin surface—nearly all women who receive radiotherapy for breast cancer experience some degree of dermatitis. The acute phase of radiation dermatitis is predominantly defined as occurring within 30–90 days of radiation exposure,^4^ and according to the Radiation Therapy Oncology Group (RTOG) toxicity criteria, it ranges from faint erythema (grade1) to dry or wet desquamation and rarely, it may progress to deep ulceration and necrosis (grade4).^5^

Risk factors increasing the development of acute breast dermatitis have been carefully studied. Larger breast size, high body mass index (BMI), texture and types of clothing items, excessive perspiration, and radiosensitivity resulted from DNA repair disorders are major patient‐related factors, along with treatment‐related factors such as utilizing two‐dimensional (2D) techniques, dosimetry, and position.^4^, ^6^. The use of low energy photons would increase the risk as well but nowadays almost all treatment facilities use 6MV photons.

Despite remarkable development in radiotherapy techniques, effective preventive interventions are still lacking, and current evidence does not provide adequate guidelines for managing acute skin reactions.^7^ Historically, recommendations for the prevention and treatment of radiation dermatitis were based on the personal and traditional experiences of radiation oncologists, nurses, or patient experiences. While the available literature does not clearly define the treatment of choice for radiation dermatitis based on high‐level evidence, it is mandatory to note that recommendations used in practice have not been found to cause any harm or negative interaction with RT.^4^ Various topical treatments have been examined in clinical trials, such as aloe Vera gel, hyaluronate creams, Calendula, petrolatum, sucralfate cream, Curcumin gel, Chamomile gel, and dressings; but the results were conflicting in reducing skin symptoms.^7^, ^8^, ^9^ Also, recent publications recommend the use of topical corticosteroids.^7^, ^10^ Overall, in most institutions, physicians prescribe topical agents, such as Aquaphor, aloe Vera, or silver sulfadiazine after the onset of evident skin alterations. Although up to 50 percent of patients who suffer from radiation dermatitis take alternative medicines, including botanical preparations, commonly combined with different traditional drugs.^11^, ^12^ According to a study by Nazarian et al., approximately 15 percent of radiation oncologists across the USA refer radiation dermatitis patients to dermatologists, and it is even recommended to take a multidisciplinary approach more frequently.^13^

Self‐treatment has become somewhat common in developed countries, and it is even more common in developing countries. In developing countries, people often use OTC products as self‐medicine and prescription medicines without supervision.^14^ Most patients prefer self‐treatment over seeking help from physicians and health care centers for cultural and financial reasons.^15^ Many patients also use opiates and illegal substances as a traditional home remedy. Regardless of the specific drugs used as self‐medication, the literature points to a high prevalence of unsupervised and non‐rational use, reporting over 50 percent of medications are being misused.^17^ According to a study in 2019, the prevalence of self‐medication is high amongst Iranian women (approximately 75 percent had a history of self‐medication use), and two of the most important reasons were the perception that self‐medication is harmless and the availability of medications at home.^16^ Besides that, self‐medication in dermatology has increased during the COVID‐19 pandemic. Choudhary et al. reported that self‐medication for dermatologic situations has increased to 48 percent compared to 15 percent before lockdown. Fear of acquiring infection from health care centers through multiple visits is one of the contributing factors.^18^

There are relatively few detailed pharmacological and clinical data about adverse drug reactions due to self‐medication. A study by Berreni et al. has shown that self‐medication‐related adverse drug reactions are frequently reported to pharmacovigilance centers, as they represent 1.5 percent of the registered adverse drug reactions. They are ‘serious’ in almost three‐fourth of cases and happen most frequently in women.^19^

In this report, our patient attempted to treat a dermatologic condition using topical application of opium latex residue, traditionally harvested. Opium poppy (*Papaver somniferous*) is one of the earliest plants used in domestic medications. The medicinal interest came from the fact that it contains biologically active alkaloids (analgesics like morphine, muscle relaxing like papaverine, etc.). Latex is the fluid that exudes when the plant's capsule is injured. After exposure to air, the latex polymerizes and transforms into opium. The latex contains various complex proteins. Also, enzyme assays have confirmed the presence of cell‐wall‐degrading enzymes in latex serum.^24^, ^25^

The topical application of opioids has been explored in studies to reduce pain associated with cutaneous wounds. However, the available data are conflicting. It was shown that topical morphine application significantly reduced the number of myofibroblasts and macrophages in closing wounds, limiting the topical application of opioids as an analgesic therapeutic strategy to treat painful cutaneous conditions. In contrast, it was reported that activation of the δ‐ opioid receptors destabilizes intercellular adhesion and promotes the migratory keratinocyte phenotype, which contributes to fast wound closure.^20^, ^21^, ^22^ According to Stein et al., opioids are promising agents for modulating tissue and simultaneous targeting of multiple upstream molecules that cause inflammation. In addition, opioids can take advantage of natural anti‐inflammatory mechanisms, but larger clinical trials must examine whether opioids interfere with wound healing by inhibiting cytokine release. Also, they declare that in contrast to currently available anti‐inflammatory agents (steroids, NSAIDs, biologics) or local anesthetics, peripherally acting opioids carry little risk of organ toxicity or infection.^23^ The latter claim may relate to pharmaceutical opioids on epithelialized surfaces, though it might not be the case for opium latex extract (containing enzymes) on a de‐epithelialized skin, similar to our patient. Additionally, traditionally extracted opium latex may contain impurities, fungi, and bacteria concerning infection concern.

It is mentioned in anesthesiology that transdermal drug absorption and systemic effect generally require high solubility in water and oil, low molecular weight, and high potency, as seen in Fentanyl, or the existence of a de‐epithelialized skin area for other opioids.^20^ Fentanyl is the prevalent transdermal opioid and has been thoroughly studied as a cornerstone in opioid prescription. Data show that along with its pharmaceutical features, dermal variables do affect the rate of transdermal fentanyl absorption. For example, differences in skin thickness and degree of keratinization will alter the systemic bioavailability and account for the great interindividual variability reported with transdermal opioid absorption.^26^ Exposed tissue lacking a stratum corneum, such as mucosa, is shown to have a more than 30‐fold increase in absorption.^27^ Skin temperature elevation augments the absorption of transdermally applied Fentanyl, theoretically due to cutaneous vasodilation or enhanced solubility of the drug.^26^ Even though most fatal fentanyl overdose cases result from deliberate abuse or suicide,^29^ case reports detail that elevation in the skin or the surrounding temperatures from sources such as hot tubs, or heating blankets may lead to fentanyl overdose.^26^, ^28^ An increase of the skin temperature from 32 to 40°C is reported to cause a gradual increase of cutaneous blood flow 10 to 15 times that of control, as measured by Doppler flowmetry.^28^ Studies have been performed to evaluate changes in skin temperature after radiation therapy.

Interestingly, a recent study by Zhu et al. demonstrated that in comparison to the baseline, the difference of average and maximum skin temperature increased significantly over time during radiotherapy (*p* <.05). The onset of this increase was accompanied by radiation dermatitis, and the maximal temperature change also appeared at the peak of Radiation Therapy Oncology Group (RTOG) scores.^30^ All the factors mentioned above may have their specific role in the culmination of our patient's home remedy with opioid overdose symptoms and emergency admission. Furthermore, the existing moist environment (tissue hydration) and the occlusive nature of the latex residue applied on the skin possibly contributed further to both systemic absorption and skin infection.^31^

## 4 CONCLUSION

Observation of this case and similar self‐treatments in clinics demonstrates the need to pay more attention to patient education regarding the complicated nature of radiation dermatitis (particularly during the COVID‐19 pandemic) and seeking effective treatment consensus toward a standard approach. It is also important to warn patients about applying topically on a de‐epithelialized skin as the consequences might be extraordinary and sometimes life‐threatening.

## CONFLICTS OF INTEREST

Authors declare that they have no conflicts of interest.

## AUTHOR CONTRIBUTIONS

S.A.J., O.M. and D.S. contributed in conception, design and drafting of the manuscript. J.S.W. and M.D, contributed in data collection. E.Z., S.M.H., S.M., and D.F. contributed in drafting of the manuscript. S.A.J. supervised the study. All authors approved the final version for submission.

## ETHICAL APPROVAL

The study was approved by Sabzevar University of medical Sciences. The study conforms to recognized standards is of Declaration of Helsinki. An informed written consent form was obtained from patient (IR.MEDSAB.REC.1400.043).

## Data Availability

The data sets used and/or analyzed during the current study are available from the corresponding authors per request.
